# The Role of Adipose-Derived Stem Cells in Breast Cancer Progression and Metastasis

**DOI:** 10.1155/2015/120949

**Published:** 2015-04-27

**Authors:** Riccardo Schweizer, Wakako Tsuji, Vijay S. Gorantla, Kacey G. Marra, J. Peter Rubin, Jan A. Plock

**Affiliations:** ^1^Department of Plastic Surgery and Hand Surgery, University Hospital Zurich, 8006 Zurich, Switzerland; ^2^Adipose Stem Cell Center, Department of Plastic Surgery, University of Pittsburgh, Pittsburgh, PA 15261, USA; ^3^McGowan Institute for Regenerative Medicine, University of Pittsburgh, Pittsburgh, PA 15261, USA; ^4^Department of Surgery, Shiga Medical Center for Adults, Shiga 524-8525, Japan; ^5^Department of Bioengineering, University of Pittsburgh, Pittsburgh, PA 15261, USA

## Abstract

Conventional breast cancer extirpation involves resection of parts of or the whole gland, resulting in asymmetry and disfiguration. Given the unsatisfactory aesthetic outcomes, patients often desire postmastectomy reconstructive procedures. Autologous fat grafting has been proposed for reconstructive purposes for decades to restore form and anatomy after mastectomy. Fat has the inherent advantage of being autologous tissue and the most natural-appearing filler, but given its inconsistent engraftment and retention rates, it lacks reliability. Implementation of autologous fat grafts with cellular adjuncts, such as multipotent adipose-derived stem cells (ADSCs), has shown promising results. However, it is pertinent and critical to question whether these cells could promote any residual tumor cells to proliferate, differentiate, or metastasize or even induce *de novo* carcinogenesis. Thus far, preclinical and clinical study findings are discordant. A trend towards potential promotion of both breast cancer growth and invasion by ADSCs found in basic science studies was indeed not confirmed in clinical trials. Whether experimental findings eventually correlate with or will be predictive of clinical outcomes remains unclear. Herein, we aimed to concisely review current experimental findings on the interaction of mesenchymal stem cells and breast cancer, mainly focusing on ADSCs as a promising tool for regenerative medicine, and discuss the implications in clinical translation.

## 1. Introduction

Breast cancer is the most-frequently diagnosed cancer and a leading cause of cancer-related death in women worldwide [1–3]. Great effort has been put into pursuing the understanding of breast cancer development, progression, and invasion, as well as implementation of appropriate therapies. Depending on breast cancer stage, therapy may include chemotherapy, irradiation, and, most frequently, surgical treatment ranging from local excision and lumpectomies to modified and radical mastectomies. Oncological surgery is disfiguring and the original anatomical contours of the breast often require reconstitution. Besides the use of synthetic prosthetics or flap surgery, a more recent alternative for restoring the breast shape and camouflaging scars is transplantation of autologous lipoaspirates, referred to as “lipofilling” or “fat grafting.” Ideally, autologous fat transplantation has the advantage of providing a more natural appearance after reconstruction, in addition to being readily available tissue coupled with low donor-site morbidity from liposuction as compared to flap surgery [4]. However, long-term outcomes are unpredictable in terms of engraftment of transplanted fat aliquots, as there is a variable loss of volume, which often dictates unsatisfactory final outcomes and the necessity for repetitive lipofilling sessions [5–7]. The reason has mainly been attributed to poor vascularization of fat grafts with consequent fat necrosis and/or apoptosis [5]. To overcome this drawback, supplementation with adipose-derived stem cells (ADSCs) isolated from white adipose tissue (WAT) has been proposed, which is believed to improve fat engraftment [5, 7–9] and have additional positive effects on scars and damaged skin after irradiation therapy [10, 11]. These cells are incorporated in the autologous fat graft but can be isolated to further enhance the regenerative potential of smaller volume injections.

ADSCs share similarities with mesenchymal stromal cells (MSCs) isolated from bone marrow (BM-MSCs) [12]. Through cytokine and growth factor release, ADSCs have shown several beneficial effects in inflammatory and autoimmune diseases and ischemic conditions [13–15]. Moreover, inherent advantages over MSCs isolated from other tissues, such as higher yields and lower harvest site morbidity [16], as well as their natural relation to WAT itself, make ADSCs an ideal tool for soft tissue reconstruction. Early reports show beneficial effects of ADSCs on autologous fat grafting with improved retention rates when coinjected [4, 9, 17–19].

MSCs are able to home to sites of tissue injury and inflammation [20], as well as the cancer microenvironment (CME) [21, 22]. In this regard, some authors proposed the use of MSCs either as a vector for anticancer therapy or as an adjunct treatment for increasing cancer cell susceptibility to chemotherapies [23, 24].

However, both BM-MSCs and ADSCs are also suspected to promote tumor development and progression, as well as recurrence in different cancer types [25–27]. MSCs in general have controversially been reported to support [26, 28–31] or to suppress [32–34] cancer cells. Thus, considering the fact that the risk of breast cancer recurrence is up to 13% after adjuvant therapy [35], investigating the effects of ADSCs on breast cancer prior to performing ADSC-enhanced fat grafting for reconstructive purposes after oncological surgery on a routine basis is of the utmost importance.

Several mechanisms have been proposed through which ADSCs, and more in general MSCs, interact with cancer cells and influence their microenvironment. These include paracrine signaling and cell-to-cell signaling, as well as differentiation into cancer-associated myofibroblasts (CAFs) or incorporation into newly formed vessels, leading to morphological and functional alterations of both cancer cells and MSCs in a bidirectional manner and the cancer niche itself [36–38]. Furthermore, several reports on the interaction between ADSCs and breast cancer cells (BCCs) have been published [30, 38–43].

The use of ADSCs for reconstructive purposes after breast cancer surgery has gained attention in recent years. The effects of ADSCs, which might improve fat retention after soft tissue reconstruction, potentially could be beneficial for the survival and promotion of residual cancer cells, a sort of “double-edged sword.” In this review, we will focus on the influence of ADSCs on BCCs and concisely summarize different putative mechanisms potentially involved in promotion and spread of breast cancer and discuss the difference to actual clinical findings.

## 2. Adipose-Derived Stem Cells

Just over a decade ago, Zuk et al. reported a multipotent, undifferentiated, self-renewing progenitor cell population isolated from WAT that is morphologically and phenotypically similar to BM-MSCs [12]. ADSCs were found to be able to differentiate into a variety of mesenchymal lineages including adipogenic, osteogenic, chondrogenic, and hepatocytic differentiation [12]. Through paracrine secretion of a broad selection of cytokines, chemokines, and growth factors, ADSCs have been shown to have antiapoptotic, proangiogenic, anti-inflammatory, immunomodulatory, and antiscarring effects. This potential makes them promising candidates for cellular therapy in regenerative medicine [9, 15, 44, 45]. Unlike bone marrow, fat is abundantly available and easily accessible through liposuction and can yield significantly higher amounts of cells, which makes adipose-derived cells appealing for regenerative medicine [14].

### 2.1. Sources and Subpopulations of ADSCs

The most common source for ADSCs is abdominal fat [23, 46, 47], as well as breast tissue, either after reduction mammoplasty [42, 48, 49] or after breast cancer surgery [50, 51]. The surgical technique and the back-table processing after harvesting are not discussed in detail in the reviewed papers. Thus, any comparisons between studies that will facilitate standardization of such parameters remain a challenge. It has been shown that the anatomical location of harvest can influence proliferation and function [52], differentiation ability [53], and apoptotic susceptibility [54] of ADSCs. For example, ADSCs derived from superficial abdominal fat depots [54] are more resistant to apoptosis, which might be relevant to ADSC survival in the highly active tumor microenvironment. Indeed, ADSCs from different anatomical regions (e.g., inguinal, omental, and pericardial) have been found to express different surface marker patterns [55] and the cell yields of ADSCs also vary by anatomical region of isolation [56].

Breast ADSCs seem to express similar surface marker phenotypes as abdominal ADSCs (positive for CD29, CD73, CD90, and CD105 and negative for CD14, CD31, CD34, and CD45) according to a recent report by Hanson et al. [57], although CD34 expression was found to differ between breast ADSCs isolated from cancer-affected mammary fat and normal breast fat tissue [58]. Regardless of the passage, ADSCs derived from normal breasts were CD34^+^, in contrast to CD34-negativity in cancer afflicted breast tissue-derived ADSCs. This is in contrast to a recent report by Yang et al., which found only minimal expression of CD34 in normal breast-derived ADSCs [48]. Nevertheless, ADSCs from abdominal and normal breast fat share similar genetic profiles [59]. Moreover, reports of ADSCs isolated from primary breast cancer tissue have been published [50, 60–62].

After homogenization of whole fat or lipoaspirates, a pooled cell pellet, the stromal vascular fraction (SVF), remains. The SVF contains a heterogeneous population of cells that includes at least four subpopulations with distinct surface marker phenotypes, in addition to erythrocytes and lymphocytes, namely, endothelial progenitor cells (EPCs; CD45^−^CD31^+^CD34^+^), mature endothelial cells (ECs; CD45^−^CD31^+^CD34^−^), pericytes (CD45^−^CD31^−^CD34^−^CD146^+^), and supra-adventitial ADSCs (CD45^−^CD31^−^CD34^+^) [63, 64]. Adipose-derived pericytes are rare (~1% of SVF) and are thought to be a progeny for the less primitive ADSCs, which express mesenchymal surface markers such as CD73, CD90, and CD105, but also CD34 [65]. However, the International Society for Cell Therapy (ISCT) definition for plastic adherent MSCs clearly includes the absence of CD34 [66]. Nevertheless, both pericytes and ADSCs have excellent adipogenic differentiation potential, which makes them both ideal cells for reconstructive purposes [63, 64]. CD34^+^ cell prevalence in fat grafts correlates with extent of graft retention and shows individual variability among patients [9]. In a joint statement paper from the International Federation for Adipose Therapeutics and Science (IFATS) and the ISCT, ADSCs were defined as a CD34^+^ subpopulation of the SVF [67].

Among the different studies investigating ADSCs and BCCs, there is a consensus that ADSCs express mesenchymal surface markers such as CD29, CD44, CD73, CD90, and CD105 and lack hematopoietic and endothelial markers (e.g., CD31, CD45). However, reports of CD34 expression are conflicting, with different authors naming “ADSC” cell populations either with or without CD34 expression. One should keep this in mind when comparing experimental results, since different expressions of CD34 could mean different cell subpopulations, in addition to the potential effects of culture on surface marker phenotype switch or loss [51]. Different cell subpopulations of the SVF are likely to share similarities and overlap in some surface marker expression but might have slightly different differentiation potential and/or functional characteristics [44]. The translational relevance of CD34 expression currently remains unclear.

In this review, ADSCs will be generally termed as those adipose-derived cells which are plastic adherent and can be expanded in culture after isolation of the SVF. This comprises cells that present a heterogeneous expression of CD34 but express unquestionable mesenchymal markers (e.g., CD29, CD44, CD73, CD90, and CD105) and lack hematopoietic and other endothelial markers (e.g., CD31, CD45).

## 3. Breast Cancer Cell Lines

Table 1 gives an overview of the BCC lines most commonly used for experimental studies on ADSCs and breast cancer interaction [68, 69]. Mainly, most of the experiments make use of human cell lines in humanized murine (xenotransplant) models* in vivo*. MCF-7 is the most common cell line used, especially to assess driving mechanisms of breast cancer progression from a relatively low malignancy to an invasive and metastatic phenotype [36, 42, 46, 47, 50, 70, 71]. The MDA-MB-231 line, on the other hand, is mostly used to investigate metastatic spread and basic biology of aggressive breast cancers [22, 30, 42, 72–74]. The different cell lines have distinct characteristics in culture and* in vivo* and may be used for specific research aims, so MCF-7 and BT-474 cells are ideal for investigation of hormone-receptor roles and their associated therapeutic approaches; ZR75.1 and SKBR3 cells, both HER-2 positive, might be used for testing therapies similar to trastuzumab; MDA-MB-231 and MDA-MB-436 are used for research on “triple-negative,” basal-like breast cancers [69].

Many of these cell lines were isolated many decades ago and were immortalized, with changes to both gene expression and phenotype over time as a potential consequence. A number of commonly used cell lines such as MCF-7 were isolated from metastatic pleural effusions (MPEs) and might not depict the most common tumor biology but an advanced one, due to originating from a metastatic cancer.

## 4. Interactions between Adipose-Derived Stem Cells and Breast Cancer Cells

### 4.1. ADSC Homing and Migration

There is evidence that MSCs home to injured tissue, sites of inflammation, and tumor niches [20, 21, 75]. This has been shown* in vivo* when administered intravenously and also for endogenous MSCs [76]. Tumor irradiation also promotes MSC recruitment into the irradiated area, probably due to induced tissue inflammation [77] or the necessity for tissue repair. Due to their inherent ability to home to cancer tissue as well as hinting to sensitizing cancer cells for chemotherapy, MSCs have also been proposed as vehicles for targeted anticancer drugs or gene therapy [49, 78, 79].

There are a multitude of surface signaling molecules, cytokines, and chemokines that are able to induce and control MSC recruitment and migration from their physiological niches and their homing into the injured tissues and cancer. Granulocyte colony stimulating factor and granulocyte-macrophage colony stimulating factor are two of the most well-known factors widely used for stem cell mobilization in the clinical setting [80, 81]. Stromal-derived factor 1 (CXCL-12) and its receptor CXCR-4 are also key players in cellular homing [82, 83] and have been shown to be involved in MSC migration, in addition to having an important role also in (tumor) angiogenesis [84]. Other molecules, for example, vascular cell adhesion molecule 1, MCP-1, and MMPs, are also involved in the complex and multifactorial MSC homing process [85–87].

ADSCs as a component of WAT are physiologically located in the breast and potentially near any occurring breast cancer. Moreover, additional ADSCs could be inoculated through reconstructive cell-assisted lipografting close to the cancer bed. This is different than BM-MSCs, which must be recruited through mobilization from the bone marrow into circulation and home to cancer. An interesting study by Kidd et al. suggests that mobilization from both fat and bone marrow may be induced by breast cancer, with the two cell types playing distinct roles in the CME [76].* In vitro*, ADSCs have been found to migrate towards conditioned medium (CM) of both MDA-MB 231 and 4T1 breast cancer cells [88].

Karnoub et al. observed homing of intravenous-applied MSCs to the tumor niche, with no evidence of accumulation in filtering organs [26]. This agrees with another work showing viable GFP^+^ ADSCs in breast cancer tumors after two weeks in a perivascular location [89] after homing. Other reports indicate substantial engraftment of human MSCs in the liver in addition to being present in tumor tissue for weeks [90]. Regardless of local or intravenous delivery, they promoted both tumor growth and invasiveness [30]. Direct coinjection of ADSCs and BCCs increased growth to a higher extent, suggesting a partial entrapment of injected cells in filtering organs (e.g., lungs, spleen, and liver). ADSCs within the tumor survived for at least 20 days and were found to differentiate into ECs and incorporate into new cancer-associated vasculature. In Karnoub's study, metastases were increased under the influence of MSCs for several BCC lines, including high malignant MDA-MB-231 and low malignant MCF-7 cells. This effect was abolished when MSCs were injected in the mammary pad contralateral to developing breast cancer, unlike results of another report, where, interestingly enough, cells injected subcutaneously were able to home to the tumor site on the contralateral mammary pad through blood circulation [89], underscoring the ability of MSCs to home to sites of tissue damage following different paths.

### 4.2. Cancer Promotion and Suppression

Studies investigating the impact of ADSCs, and more in general MSCs, on cancer growth dynamics and patterns, as well as progression to metastatic disease, revealed somewhat contradictory results, showing both promoting and suppressing effects. Tables 2 and 3 summarize the most important experimental* in vitro* and* in vivo* studies, respectively.

In their 2007 study, Karnoub et al. reported that BM-MSCs promote the disposition of BCCs to migrate when cocultured with low malignancy cell lines such as MCF-7 [26]. A number of preclinical studies followed, suggesting that BM-MSCs can exert a promoting influence on the growth and spread of breast cancer [29, 31, 91, 92]. In 2009, the first reports showing similar cancer-promoting effects with the use of ADSCs were published, depicting that the issue might extend to MSCs coming from different sources as well [38, 73]. In a similar fashion, Kucerova et al. found that BM-MSCs and ADSCs promoted proliferative effects in a variety of BCC lines [93], but not on SKBR3 [23]. This was in line with reports from a Chinese group, which found decreased tumor proliferation with high numbers of MSCs [34, 94]. Sun et al. also showed that human BM-MSCs and ADSCs homed to tumors and were able to inhibit growth of high malignancy MDA-MB-231 cells and decrease metastatic spread of a normally migratory cell line* in vivo* [49]. These findings were confirmed in later studies by the same group with both umbilical cord-derived MSCs and ADSCs injected simultaneously with or three weeks after inoculation of BCCs [95]. This might be a very important finding, as the timing might more appropriately reflect the clinical scenario of stem cell-enhanced autologous fat grafting.

Rowan and colleagues discovered that ADSCs did not increase proliferation in triple-negative BCCs but did slightly in hormone-receptor positive cells such as MCF-7 and BT-474. On the other hand, not only the* in vitro* migration potential of triple-negative MDA-MB-231 was enhanced by ADSCs, but also their CM was enough to achieve similar results, suggesting a paracrine mechanism [71]. These results are similar to those observed with the bone marrow-derived counterpart in the earlier report by Karnoub et al. [26].

Of WAT-derived cells, CD34^+^ cells seem to be at least partly responsible for tumor-promoting ability, as they increased tumor sizes significantly when coinjected with BCCs. In addition, CD34^+^ cells seem to be more efficient in a metastatic shift of triple-negative MDA-MB-436 and HCC1937 cells in a murine xenograft model [43]. In a study published later by the same group, two distinct CD34^+^ populations were found to act in concert when promoting breast cancer growth [41]. EPCs promoted neovascularization to a higher extent and were more prone to migration into lymph nodes and metastasis formation, whereas ADSCs locally promoted tumors more than EPCs. Strikingly enough, CD34^−^ cells promoted growth to a lesser extent, and metastases were similar to controls without WAT cells. Ironically, the CD34^+^ subpopulation is the one which shows high benefits for retention of fat grafts and therefore would be an appealing tool for reconstructive efforts [9].

Noteworthily, two published papers by Ke et al. and Zimmerlin et al. included* in vivo* models in which they seeded cancer cells in numbers as low as ten and 100 cells, respectively [5, 96]. The first group showed that ten murine 4T1 breast cancer cells (low malignancy) were able to grow into a tumor and metastasize upon coinjection with murine BM-MSCs, whereas the same BCCs alone failed to do so [96]. The authors suggested increased angiogenesis, as depicted by enhanced vascularity next to GFP^+^ BM-MSCs as one of the mechanisms. Interestingly enough, and in contrast to other studies, MSCs were not present in the tumor at later time points beyond 11 days [96]. Zimmerlin et al. isolated cells in different dormancy states: persistent, dormant cells after surgical therapy and active cells representing the active disease as a primary or recurrent tumor. The authors isolated mainly three cancer cell types, namely, small resting and large active cancer cells, both CD90^+^ and a third CD90^−^ population. Small resting cells were rare and represented only a small portion of the isolated cells. However, these cells may potentially lead to recurrence [5]. Combined with ADSCs, 100 small resting cells were not affected. The same aliquot of large cells was not capable of developing a cancer nodule but developed to a significant size when coinjected with ADSCs. These findings could be explained by the autonomy of slow-growing dormant cells, whereas active cells require a high amount of growth factors and good vascularity. This is in line with other findings in which breast ADSCs were able to promote the progression and invasion of the invasive cancer cell line T4-2, but not its preinvasive variant HMT-3522 S3 [74]. These results suggest that fat grafts supplemented with ADSCs for reconstruction could be used in patients after complete and terminated cancer-therapy and documented healing, since they may affect active but not resting cancer cells [5].

### 4.3. MSCs and the Cancer Microenvironment

Besides being a highly proliferative and dynamic mammary gland tissue, breast tissue contains a stroma with a heterogeneous cell population including adipocytes, myofibroblasts, MSCs, and ECs, as well as macrophages and other immune system cells [97]. Similarly, this stroma is actively involved in creating the CME, which is composed of highly proliferative malignant cancer cells and several nonmalignant elements including cancer-associated vessels, the extracellular matrix (ECM), CAFs [36, 76], stromal cells such as MSCs [98], and immune cells like macrophages and lymphocytes [99]. Emulating a chronic wound and secreting chemoattractant factors, tumors “trick” and attract MSCs from the bone marrow and possibly other locations such as local and peripheral fat [100].

The interaction between the stroma resident cells such as ADSCs and cancer-associated fibroblasts and primary cancer cells is sophisticated and happens in a bidirectional fashion, with the different cells influencing each other on different levels. MSCs that have homed to a tumor can have different fates: they may survive and exist as MSCs or differentiate into another cell type, such as ECs, pericytes, or CAFs [101–103]. MSCs and CAFs share similarities in regard to phenotype and surface markers, but CAFs additionally express fibroblast-specific protein and fibroblast activation protein, as well as *α*-SMA, and have been shown to produce higher levels of IL-4, IL-10, TGF-*β*1, and VEGF [104]. The basal-like CD44^+^CD90^+^ small cells at the stroma/tumor interface cross talk with surrounding CAFs, which provides an ideal niche for the growing tumor mass. Those cells later migrate to the inside of the tumor bulk and become highly proliferative CD44^+^90^−^ cells. Noteworthily, the CD44^+^90^+^ cells have been regarded as tumor cell progenitors and might serve as cancer stem cells (CSCs) [40].

BM-MSCs and ADSCs have been shown to differentiate into CAFs* in vivo *and* in vitro* [103, 105–109]. Kidd et al. found that CAFs originate mainly from endogenous bone marrow precursor cells, whereas progenitor cells from local adipose tissue are the origin of pericytes and ECs involved in the growing cancer vascular network and constitute the majority of the recruited cells [76]. Also, to their advantage, CAFs can be activated by BCCs, leading to increased tumor growth [84].

ADSCs in culture with CM from MDA-MB-231 and MCF-7 tumors partly differentiated into myofibroblasts and promoted cancer invasion ability* in vitro* through a TGF-*β*1/Smad dependent pathway [37]. This depicts bidirectional signaling, reciprocal influence, and consequent phenotype modifications between ADSCs and BCCs [36]. Breast carcinomas often involve a desmoplastic reaction similar to the one found during the healing process in wounds [74]. The EGF/EGFR/Akt-dependent pathway was shown to be involved and the promoting effect reverted after EGF-blockade [98].

Accumulating evidence suggests that chronic inflammation, as found in tumors, is involved in the progression and recurrence of breast cancer [110]. Immune system cells can attract many other host cells, including macrophages and MSCs [111, 112]. Macrophages secrete relevant amounts of MMPs, which increase the invasion ability of cancer [110] and are able to suppress T-cell antitumor effects through a HIF-*α* dependent pathway [113]. MSCs show inhibitory effects on local immune reaction against breast cancer, with increased T reg (CD4^+^FoxP3^+^) levels in tumors and diminished natural killer cells [90]. Moreover, MSCs are activated to secrete anti-inflammatory cytokines when exposed to proinflammatory cytokines in the tumor milieu, which enables tumor immune evasion [104, 114]. ADSCs isolated from breast cancers also secrete high levels of immunosuppressive cytokines such as IL-4, IL-10, and TGF-*β*1 [61].

### 4.4. Cytokines, Chemokines, and Growth Factors: The Influence of Paracrine Signaling versus Cell-to-Cell Contact

To shed light on further mechanisms besides endocrine- and hormone-dependent pathways, several studies have addressed the question of whether cell-to-cell contact promotes breast cancer progression under ADSC influence [23, 46, 58, 88, 104, 115]. ADSCs are known to secrete growth factors, cytokines, and chemokines [15, 93]. Indeed, several factors are increasingly present in the CME, including HGF, IL-6, IL-8, SDF-1, TNF-*α*, TGF-*β*1, and VEGF [39, 104]. However, their specific role in breast cancer is still poorly understood, even though some of the mediators such as IL-6 and TGF-*β*1 seem to be clearly involved in progression of breast malignancies into a more malignant phenotype [39, 106, 116].

In their 2011 published work, Kucerova et al. found that ADSC-CM increased BCC proliferation in a dose-dependent manner, suggesting a cell-to-cell contact-independent mechanism. The CM contained high levels of IL-6, IL-8, MCP-1, and VEGF [93]. Strikingly, coculture of BCCs with CM was more potent in promoting proliferation than direct coculture of the cells. In a recent work, the same authors further investigated the paracrine effects of ADSCs on the triple-negative cell line SKBR-3 and found CM to induce epithelial-mesenchymal-transition and mammosphere formation, as well as increased cell motility [23]. On the other hand, interestingly, chemosensitivity of BCCs to anticancer drugs was increased by ADSC-secreted factors, which might yield to a potential adjunct for chemotherapeutic protocols. Others found that exosome-mediated cell-to-cell contact was a necessary step for ADSCs to increase tumor cell proliferation [58, 117], with activation of the Wnt pathway as a putative mechanism [46].

### 4.5. Obesity: Increased ADSC Pool

Obesity is a common condition and has been associated with increased lifetime risk of breast cancer development [118–121]. This has been linked to increased levels of aromatases in WAT and raised levels of estrogen. Surplus adipose tissue worsens the prognosis at onset of breast cancer disease and can contribute to drug resistance [72, 122]. Besides providing energy storage, fat tissue is also regarded as an endocrine organ [123]. In fact, in postmenopausal women, fat remains the most important estrogen production site [124]. WAT is also largely present in the breast and exerts both paracrine and endocrine actions on the mammary gland, as well as any developing BCCs. Leptin, IL-6, TNF-*α*, IFG, and other hormones are upregulated in obese women and contribute to a state of “chronic inflammation” [44, 125], which can promote breast cancer growth [126]. Indeed, IL-6 has been shown to promote invasion capability and is a marker for poor outcome in breast cancer patients [39, 42, 127–129] and increased IL-6 serum levels are associated with increased metastatic spread [130].

Obesity increases the overall availability and circulating number of ADSCs [44]. Overweight mice have higher yields of ADSCs in the blood stream [22]. ADSCs in obese mice differentiated more frequently into tumor-associated adipocytes and promoted tumor growth [39]. In a different setting, BCCs inhibited adipogenesis of ADSCs, which, in turn, responded with increasing proinflammatory signals, rearranging the ECM [36]. However, it is still unclear whether these findings have any relevance in the clinical setting.

Leptin found in obese patients promotes macrophage differentiation, increasing proinflammatory and proangiogenic factor secretion. In a positive feedback loop, increased proinflammatory cytokines increase the amount of preadipocytes, blocking their maturation to adipocytes, which again raises the amount of inflammatory cytokines and leptin levels [131]. This sort of interplay is believed to be able to predispose a patient to malignancy development [72]. The paracrine mechanism for matrix metalloproteinase (MMP)-2, MMP-9, and Twist1 expression is estrogen- and leptin-dependent [72]. Leptin level also correlated with higher recurrence rates in estrogen- and progesterone-receptor positive (ER^+^/PR^+^) cancers, underscoring its role in increased invasiveness [72, 132]. In addition, as shown by Rhodes et al., BM-MSCs promoted the growth of breast cancer estrogen-independently [29]. The lack of hormone receptors on basal-like BCCs such as MDA-MB-231 and SKBR-3 advocates for the hormone-independent promoting effect of ADSCs in this type of breast cancer. Interestingly, ADSCs from nonobese people had less influence on BCC proliferation [72].

## 5. Effects of ADSCs on Migration and Metastatic Spread

The spread of breast cancer to distant locations as well as cancer recurrence worsens prognosis and patient survival drastically and eventually accounts for most breast cancer-related deaths [133]. To metastasize, cancer cells need to go through a process, including invasion, migration through stroma, extravasation, and engraftment in a remote, new niche [134]. This happens directly into adjacent skin and muscle or indirectly through the lymphatic system or blood stream. Frequent distant metastasis sites are bone, brain, lung, and liver [133]. Bone marrow in the skeleton has been attributed to the promotion of growth of breast cancer metastases, due to the presence of a heterogeneous marrow stroma including MSCs, EPCs, hematopoietic stem cells, and fibroblasts among other types of cells, creating a particularly suitable environment for proliferation. Thus, it is essential to investigate and shed light on the effects that fat transplantation to the breast and, more specifically, comprised or implemented ADSCs might have in promoting breast cancer invasion and progression. Several publications report the potential enhancing effect of ADSCs on the metastatic sequence of breast cancer [30, 39, 41, 42, 49, 71].

### 5.1. ADSCs Influence on Invasion and Migration

A multitude of ADSC-secreted factors are potentially able, alone or in combination, to induce enhanced migration and invasiveness of breast cancer cells. IL-6, IL-8, MCP-1, RANTES, SDF-1, TGF-*β*1, and VEGF, among others, can shift BCCs to a more aggressive cancer phenotype, resulting eventually in increased metastatic occurrence [5, 93, 135].

SDF-1 is one important factor involved in the spread of BCCs [31, 135]. Blocking CXCR-4 receptors significantly revert the effect, even in the presence of BM-MSCs [31]. The SDF-1 pathway especially is relevant to breast cancer metastasizing to bone [136]. Importantly, CXCR-4 is also linked to poor clinical outcome in patients with breast cancer [135]. In a similar fashion, ADSCs promoted BC spread through a SDF-1-dependent mechanism both* in vitro* and* in vivo* [30]. RANTES is another relevant factor secreted by ADSCs involved in BCC migration [26, 30, 38]. Indeed, Karnoub et al. described previously that BM-MSCs produce RANTES when stimulated by BCCs, which in turn enhances their motility and favors metastasizing [26]. A similar effect could be expected for ADSCs as well. IL-6 and IL-8 are interleukins linked to increased cancer invasion and migration [73, 137, 138]. Additionally, loss of ER has been found to correlate with IL-8 upregulation and breast cancer progression in ER^−^ breast cancer cell lines [139]. Secretion of MMPs by MSCs fosters breast cancer invasion and migration through ECM modification. MMPs, a class of proteases, are involved in restructuring the tumor stroma and are increasingly expressed in the CME and believed to increase breast cancer invasion [133, 140–142]. MMP-9, for example, increases metastasis without promoting cancer growth [141]. Similarly, MMP-11 enables BCCs to migrate through complex bidirectional signaling with local adipocytes and ADSCs [140]. There is evidence that MMP-mediated BCC migration depends on interplay between the different types of MMPs and does not rely on a single MMP type [74].

### 5.2. Epithelial-to-Mesenchymal Transition

One important mechanism by which MSCs have been shown to influence cancer cells is turning premalignant or low malignant cells into an invasive and migratory phenotype through epithelial-to-mesenchymal transition (EMT) [143, 144]. EMT, known as a physiological process during development [145], has also been implicated in lung [146], prostate [147, 148], and breast cancer [23, 41, 60, 70]. During EMT, cells are unleashed from their tight junctions, allowing them to escape into the tumor/stroma complex and move through the ECM, increasing their plasticity [149]. Cell propensity for migration is increased, inducing a switch from* in situ* cancer to invasive cancer types; invasion of blood vessels and production of distant metastases are the consequences [150, 151]. MSCs can induce morphological, functional, and molecular changes in epithelial cancer cells, resulting in downregulation of epithelial-specific markers and increased migration, potentially promoting phenotype shifting and migration, generating migration-enabled CSCs, or both [70, 116, 149, 151].

Both BM-MSCs and ADSCs secrete many factors that participate in inducing EMT in breast cancer [71, 131, 152–154], and some ADSC subpopulations might be more prone to inducing EMT than others [41]. Secreted TGF-*β*1 and IL-6 especially but also IL-8 and MMPs have been long recognized to release cell-to-cell contacts of breast cancer cells and initiate metastasizing behavior [130, 149, 151, 155–157]. Additionally, hormones like leptin and osteopontin can induce EMT [158, 159].

Upon induction, typical EMT genes are upregulated by MSCs such as Slug, Snail1/2, Smad, and Twist1 [160–162]. This translates in a so-called cadherin-switch, which is a hallmark of EMT, where E-cadherin is downregulated, N-cadherin is upregulated [149, 152, 163], and mesenchymal proteins are induced (e.g., Vimentin and Actin). CAFs are also able to increase invasion and migration of luminal and basal type BCCs through the TGF-*β*1/Smad pathway [164]. Inhibition of the TGF-*β*1/Smad complex, indeed, has been found to reduce BM-MSC-mediated breast cancer progression through a repression of MSC-to-CAF differentiation [106].

Besides promoting invasion and metastasis, MSC-induced EMT might confer self-renewal activity to BCCs. Indeed, EMT might be at the basis of distant breast cancer metastatic spread, generating CD44^+^ CSCs, which are mesenchymal-like cells that can easily migrate into the blood stream and extravasate and metastasize [70, 131, 165, 166]. Moreover, while other tumor cells are more or less susceptible to anticancer therapy, CSCs seem more resistant and involved in progression to hormone-receptor negative and chemotherapy-resistant tumor cells [165, 167–170].

## 6. Clinical Implications

The concept of grafting fat to the breast for aesthetic and reconstructive purposes originated over a century ago but has been again promoted over the last twenty years [4]. While initial concerns about detection of breast cancer during screening have been refuted, the detection of ADSCs as an active component of the autologous processed graft has raised safety concerns. In general, evidence for ADSC application for breast reconstruction after cancer surgery is not voluminous. Nevertheless, several reports, mostly clinical case series, show no evidence of increased cancer occurrence after lipofilling, pointing out the importance of appropriate oncological follow-up [171–173]. In one of the biggest series of lipograft procedures after breast cancer, no increased recurrence was found during a 10-year follow-up [173], whereas higher recurrence rates were detected for* in situ* breast carcinomas after breast conserving therapy followed by autologous fat grafting in another group [174–176]. It is important to note that these reports focus on autologous fat transfer without added stem cells.

However, many reports differ with regard to patient numbers, patient selection criteria, follow-up length, and use of controls [171–173, 177]. Some case reports suggest fat grafting-related cancer recurrence, even though they fail to prove a direct link [178–180].

The first clinical study that assessed stem cell-enriched fat grafting in the postcancer scenario showed promising aesthetic results and no adverse events such as cancer recurrence [181] during a very limited follow-up period of one year. The study was criticized for only including low-risk patients and for being designed without any controls [182].

Petit et al. published a large multicenter study with a median follow-up of 19.2 months involving 513 breast cancer patients. The authors did not find that autologous fat transfer interfered with radio-oncological follow-up, but pointed out the need for further studies with a strict and long-term oncological follow-up period [174, 175]. The same authors found increased local recurrence in a case-control study of a specific subgroup of patients undergoing surgery for* in situ* neoplasias with subsequent autologous lipofilling for breast recontouring [174]. In 2013, they published an expanded study with a larger cohort and a longer follow-up period and confirmed the preliminary results, suggesting an increased cancer risk in this particular patient collective. The results might be due to an exceptionally low control incidence of local events due to selection bias [176], but other authors also agree that oncological safety could be better elucidated [183–185]. These authors did not find an increased rate of recurrence in the other patients studied, and other published case series of breast fat grafting for reconstruction have not shown an increased rate of local recurrence.

A small number of ongoing clinical trials are assessing outcomes after stem cell-enhanced fat transfer to the breast with a focus on aesthetic results (ClinicalTrial.gov; NCT01756092 and NCT01801878) and oncological results. In the GRATESC trial (NCT01035268), an ongoing prospective randomized, multicenter study started in 2010; the authors aim to investigate local and distant cancer recurrence after lipofilling for breast shape and volume improvement after breast conservative surgery, with a planned follow-up period of five years.

## 7. Discrepancies between Basic Science and Its Clinical Translation

Overall, data regarding the influence of MSC and more in particular ADSCs on breast cancer cells are controversial. A few preclinical reports show decreased breast cancer cell proliferation with high amounts of MSCs [23, 34, 94], even in highly proliferative cell lines such as MDA-MB-231 [49, 95]. On the other hand, a variety of basic science reports demonstrate a fostering effect of MSCs on breast cancer growth, progression, and metastasis [29, 36, 39, 41, 43, 60, 71]. The majority of these reports are raising concerns regarding the use of ADSC for cell-assisted fat grafting for both aesthetic and reconstructive procedures on the breast. These experimental findings are not well substantiated by clinical data thus far: there are a number of case series and one clinical study pointing out at a higher local breast cancer recurrence after lipofilling [175, 176, 178, 186]. Whether basic science is solely unmasking a potential issue, which eventually is not relevant enough to translate to clinical reality or if, indeed, ADSC-enhanced lipofilling procedures bear oncological risk, is an ongoing discussion.

This discrepancy can have several origins and definitely needs to be addressed prior to routine use of this reconstructive strategy. Many variables of the experimental setup can influence the results. As an example, in the mentioned studies, most of the utilized cells grow fast* in vitro* and form large tumors* in vivo*, which might not reflect the actual clinical reality. More likely, dormant, low active cells remain unrecognized in the tumor bed after unsuccessful surgical therapy than highly proliferative ones. Moreover, freshly isolated primary breast cancer cells from tumor excisates or MPEs [5] should be preferred for preclinical studies, along with matched fat tissue and MSCs/ADSCs from the same patient, as different donor biology can affect the MSC functionality and thus the outcome [71]. Primary breast cancer cells have been shown to have lower doubling times [187] and may have different dormancy status which has to be accounted for as well [5]. The timing of MSC addition to the tumors is another important factor. For example, injecting MSCs three weeks after BCC inoculation showed decreased metastasizing in triple-negative breast cancer [95], which is an important finding, as the chosen delayed timing might more appropriately reflect the clinical scenario of* postcancer* breast reconstruction. High amounts of tumor cells as injected in many experimental studies* in vivo* might not depict the clinical reality as well. Indeed, if residual cancer cells remain* in situ* after breast cancer surgery, it is likely that a low number of BCCs would be exposed to a significantly higher number of ADSCs supplemented to fat grafts at the time of reconstruction. Further, distinct MSC origins and species, as well as different culture conditions [50], 2D culture systems which fail to simulate the CME adequately, the BCC-to-MSC ratio, and the route of administration for* in vivo *studies are additional factors which might contribute to controversial results.

On the clinical side, the actual data has to be carefully analyzed. In our opinion, more clinical data is still needed in strong evidence for safety with the use of cell-enhanced fat transplantation. Generally, the available clinical study data suggest safe application of unprocessed autologous fat grafting. The study of Petit et al., with increased recurrent local events after fat grafting in patients with* in situ* cancer of the breast, so far is the only controlled study demonstrating an increased risk of recurrence in a specific cancer subgroup [176]. Larger controlled clinical trials are warranted and these should avoid any selection bias due to sole inclusion of a “favorable” patient population (i.e., mastectomies), which is likely to provide lower recurrence rates than expected after breast conserving surgery [183]. Additionally, large scale registries, such as the American Society of Plastic Surgeons fat grafting to the breast registry, should be broadly implemented.

## 8. Conclusions

The majority of experimental studies trend to support the propensity of MSCs and ADSCs in promoting growth, progression, and metastatic spread of residual or* de novo* breast cancer after resection. In contrast, only a few clinical case series and trials are reflective of similar findings.

Two scenarios are of interest. (1) Any residual unresected microscopic tumor foci persisting after mastectomy could be activated by ADSCs used in postsurgical restoration. (2) Occult dormant cancer cells in patients with no diagnosed breast cancer but undergoing ADSC therapies for breast augmentation may undergo a malignant transformation.

Currently, the concerns of safety and the debate on efficacy versus such unresolved risk remain ongoing until larger randomized and controlled clinical trials shed light on the scenario. Multiple recommendations based on extensive reviews are available and may be useful for patient information and selection. Overall, most of these studies do not support using autologous stem cell-enhancement at the present [185, 188–190], whereas whole fat grafting appears to be safe in many circumstances.

## Figures and Tables

**Table 1:** Most common human BCC lines used for investigation of ADSC/breast cancer interaction [68, 69, 191].

BCC line	Classification	ER	PR	Her2	In culture	Notes	References
MCF-7	Luminal A	+	±	−	Mass	Endocrine responsive Isolated from MPE	[36, 42, 46, 47, 50, 58, 61, 70–73, 98, 109]

MDA-MB-231	Basal B, claudin-low	−	−	−	Stellate	Isolated from MPE	[22, 30, 36, 38, 42, 49, 71–74, 89, 109]

T47D	Luminal A	+	±	−	Mass	Endocrine responsive Isolated from MPE	[70, 73, 93]

BT-474	Luminal B	+	+	+	Mass	Endocrine and Trastuzumab responsive Isolated from primary tumor	[70, 71]

HCC1937	Basal A	−	−	−	n/a	Isolated from primary tumor	[41, 43]

MDA-MB-436	Basal B	−	−	−	Stellate	Isolated from MPE	[41, 43]

ZR 75.1	Luminal B	+	±	+	Grape-like	Endocrine and Trastuzumab responsive. Isolated from ascites	[39, 41]

SKBR3	Luminal, Her2	−	−	+	Grape-like	Trastuzumab responsive Isolated from MPE	[23]

T4-2 (HMT-3522)	Basal B	−	−	−	Mass	Isolated from primary tumor	[74]

BC: breast cancer; BCC: breast cancer cell; ER: estrogen receptor; Her2: human epidermal growth factor receptor 2; MPE: metastatic pleural effusion; n.s.: not specified; PR: progesterone receptor.

**Table 2:** Relevant *in vitro* studies investigating the effects of ADSCs on breast cancer.

Reference	Year	ADSC origin	ADSC surface marker	BCC line	Effects on BCC
Trivanović et al. [58]	2014	Human breast (normal versus cancer-affected) and abdominal	CD44^+^CD73^+^CD90^+^CD105^+^ CD11a^−^CD33^−^CD45^−^CD235a^−^ HLA-DR^−^CD34^±^	MCF-7	Proliferation↑ (direct coculture) Proliferation↓ (indirect coculture) Different ADSCs had similar effects

Kucerova et al. [23]	2013	Human lipoaspirates	CD29^+^CD44^+^CD90^+^CD105^+^ CD14^−^CD34^−^CD45^−^	SKBR3	Proliferation↓, migration↑ EMT markers↑ BCC chemosensitivity↑

Lin et al. [46]	2013	Human lipoaspirates	CD29^+^CD44^+^CD105^+^ CD31^−^CD34^−^HLA-DR^−^	MCF-7	Proliferation and migration↑ Cell-to-cell contact needed Wnt pathway↑

Strong et al. [72]	2013	Human abdominal versus nonabdominal	n.s.	MCF-7, MDA-MB-231	Proliferation↑ Leptin/estrogen-dependent Increased effect of abdominal ADSCs from obese (versus lean and nonabdominal ADSCs)

Zhang et al. [50]	2013	Human breast (cancer-affected)	CD13^+^CD29^+^CD44^+^CD71^+^ CD105^+^HLA-I^+^ CD4^−^CD10^−^CD14^−^CD34^−^ CD38^−^HLA-DR^−^	MCF-7	Proliferation↑ Migration↑

Zhao et al. [47]	2013	Human lipoaspirates (abdominal)	CD29^+^CD44^+^CD105^+^ CD34^−^CD45^−^	MCF-7	Migration↑ Angiogenesis↓ MMPs↑

Devarajan et al. [70]	2012	Human whole fat	n.s.	4T1 (murine) BT-474, MCF-7, T47D	Proliferation↑, EMT markers↑ PDGF-dependent (paracrine)

Jotzu et al. [109]	2011	Human whole fat	CD29^+^CD44^+^CD90^+^CD105^+^ CD14^−^CD34^−^CD45^−^	MCF-7, MDA-MB-231	Migration and invasion↑ ADSCs differentiate to CAFs

Kucerova et al. [93]	2011	Human lipoaspirates	CD44^+^CD73^+^CD90^+^CD105^+^ CD14^−^CD34^−^CD45^−^	MCF-7, T47D, MDA-MB-361	BCC proliferation↑ (dose-dependent) Paracrine mechanism

Razmkhah et al. [61]	2011	Human breast (cancer-affected)	CD44^+^CD105^+^CD166^+^ CD14^−^CD34^−^CD45^−^	MCF-7	Anti-inflammatory cytokines↑ T regs↑

Yan et al. [98]	2012	Human breast (normal versus cancer-affected)	CD29^+^CD73^+^CD90^+^CD105^+^ CD166^+^ CD31^−^CD144^−^CD14^−^CD45^−^ HLA-DR^−^	MCF-7	Proliferation↑ (BC ADSCs > normal breast ADSCs) EGF/EGFR/Akt-dependent

Pinilla et al. [38]	2009	Human abdominal	n.s.	MDA-MB-231	Proliferation↑, RANTES↑ Migration↑, MMPs↑

Welte et al. [73]	2012	Human lipoaspirates (abdominal)	CD44^+^CD90^+^CD105^+^ CD11b^−^CD14^−^CD34^−^CD45^−^ HLA-DR^−^	MCF-7, MDA-MB-231, T47D	ADSC migration towards BCCs Migration and invasiveness↑ IL-8↑

ADSC: adipose-derived stem cell; BC: breast cancer; BCC: breast cancer cell; CAF: cancer-associated (myo) fibroblast; EMT: epithelial-to-mesenchymal transition; ER: estrogen receptor; Her2: human epidermal growth factor receptor 2; MMPs: matrix metalloproteinases; MPE: metastatic pleural effusion; n.s.: not specified; PR: progesterone receptor; T reg: regulatory T lymphocyte.

**Table 3:** Relevant *in vivo* studies investigating the effects of ADSCs on breast cancer.

Reference	Year	Model	ADSC origin	ADSC surface markers	BCC line	Ratio BCC/ADSC	Effects on BCC/BC
Eterno et al. [60]	2014	Mouse	Human lipoaspirates and breast whole fat (normal versus cancer-affected)	CD44^+^CD90^+^CD117^+^ CD133^+^ CD34^low^CD45^−^	MCF-7, MDA-MB-231, primary BCCs	2 : 1	No changes in MCF-7 MDA-MB-231 growth and migration↑ EMT↑ Paracrine, IL-8↑, IL-6↓

Rowan et al. [71]	2014	Mouse	Human lipoaspirates (abdominal)	CD29^+^CD34^+^CD73^+^ CD90^+^CD105^+^ CD44^low^CD45^low^	BT-474, MCF-7, MDA-MB-231	1 : 1	Tumor growth*↔* Migration and metastasis↑ EMT induction

Orecchioni et al. [41]	2013	Mouse	Human lipoaspirates	CD31^+^CD34^+^CCRL2+ CD13^−^CD45^−^ (EPC) and CD13^+^CD34^+^ CD140b^+^ CD31^−^CD45^−^ (ADSC)	HCC1937, MDA-MB-436, ZR75-1	5 : 1	Tumor growth↑ Metastatic spread↑ EMT↑ Effect of ADSCs > EPCs

Chandler et al. [36]	2012	Mouse	Human lipoaspirates	CD13^+^CD29^+^CD44^+^ CD73^+^CD90^+^CD105^+^ CD166^+^ CD14^−^CD31^−^CD45^−^	MCF-7, MDA-MB-231	1 : 1	Tumor growth↑ Angiogenesis↑ Bidirectional signaling ADSCs differentiate to CAFs

Zhang et al. [22]	2012	Mouse	Murine (endogenous)	CD34^+^ CD31^−^CD45^−^	E0771, MDA-MB-231	n.s.	Circulating ADSCs↑ in cancer ADSCs incorporate into tumor vasculature (as pericytes)

Zhao et al. [74]	2012	Mouse	Human breast (normal)	CD29^+^CD73^+^CD90^+^ CD105^+^ CD14^−^CD31^−^CD45^−^	HMT-3522 S3 (preinvasive), HMT-3522 T4-2 (invasive), MDA-MB-231	1 : 1, 3 : 2	Tumor growth↑ Tumor invasiveness↑ Angiogenesis*↔* No effect on preinvasive BCCs

Dirat et al. [39]	2011	Mouse	Murine 3T3 adipocytes	—	4T1, 67NR, (murine) ZR 75.1, SUM159PT	n.s.	Metastatic spread↑ IL-6-dependent

Martin-Padura et al. [43]	2012	Mouse	Murine whole fat	CD34^+^ CD45^−^	HCC1937, MDA-MB-436	5 : 1	Tumor growth↑ Metastatic spread↑ Angiogenesis↑

Zimmerlin et al. [5]	2011	Mouse	Human abdominal whole fat	CD34^+^CD44^+^CD73^+^ CD90^+^CD105^+^CD146^+^ CD45^−^CD31^−^	Human MPE	n.s.	Tumor growth↑ (active cells, but not resting cells)

Muehlberg et al. [30]	2009	Mouse	Murine whole fat	CD44^+^CD90^+^CD105^+^ CD11b^−^CD14^−^CD34^−^ CD45^−^HLA-DR^−^	4T1 (murine), MDA-MB-231	1 : 10	Tumor growth↑ Metastatic spread↑ Paracrine through SDF-1 ADSCs home to tumor and differentiate to ECs

Sun et al. [49]	2009	Mouse	Human breast whole fat	n.s.	MDA-MB-231	2 : 1	Tumor growth↓ Metastatic spread↓ No early carcinogenesis improvement

Walter et al. [42]	2009	Mouse	Human breast whole fat and abdominal lipoaspirates	n.s.	MCF-7, MDA-MB-231	1 : 1	Tumor migration and invasiveness↑ IL-6-dependent

Zhang et al. [89]	2009	Mouse	Murine whole fat (obese mice)	CD34^+^ CD31^−^CD45^−^ (ADSC) and CD31^+^CD34^+^ CD45^−^ (EPC)	4T1, EF43.fgf4 (murine), MDA-MB-231	n.s.	Tumor growth↑ ADSCs home to tumor (perivascular space)

ADSC: adipose-derived stem cell; BC: breast cancer; BCC: breast cancer cell; EC: endothelial cell; EMT: epithelial-to-mesenchymal transition; EPC: endothelial progenitor cell; ER: estrogen receptor; Her2: human epidermal growth factor receptor 2; MMPs: matrix metalloproteinases; MPE: metastatic pleural effusion; n.s.: not specified; PR: progesterone receptor; T reg: regulatory T lymphocyte.

## Abbreviations

ADSC: Adipose-derived stem cell
BCC: Breast cancer cell
BM-MSC: Bone marrow-derived stromal cell
CAF: Cancer-associated myofibroblast
CME: Cancer microenvironment
CSC: Cancer stem cell
CM: Conditioned medium
EC: Endothelial cell
EPC: Endothelial progenitor cell
EMT: Epithelial-to-mesenchymal transition
ER: Estrogen receptor
MMP: Matrix metalloproteinase
MSC: Mesenchymal stem (stromal) cell
MET: Mesenchymal-to-epithelial transition
MPE: Metastatic pleural effusion
PR: Progesterone receptor
SVF: Stromal vascular fraction
WAT: White adipose tissue.
